# Synovial calprotectin is a reliable biomarker for periprosthetic joint infections in acute-phase inflammation — a prospective cohort study

**DOI:** 10.1007/s00264-022-05421-1

**Published:** 2022-05-07

**Authors:** Igor Lazic, Peter Prodinger, Maximilian Stephan, Alexander T. Haug, Florian Pohlig, Severin Langer, Rüdiger von Eisenhart-Rothe, Christian Suren

**Affiliations:** 1 Department of Orthopaedics and Sports Orthopaedics, School of Medicine, Klinikum Rechts Der Isar, Technical University of Munich, Ismaninger Str. 22, 81675 Munich, Germany; 2 Department of Orthopaedic Surgery, Norbert-Kerkel-Platz, Krankenhaus Agatharied, 83734 Hausham, Germany

**Keywords:** Periprosthetic joint infection, Dislocation, Implant breakage, Post-operative, Calprotectin

## Abstract

**Purpose:**

Diagnosing periprosthetic joint infections (PJI) are challenging and may be hampered by the presence of other causes of local inflammation. Conventional synovial and serum markers are not reliable under these circumstances. Synovial calprotectin has been recently shown as a promising biomarker for PJI in total hip (THA) and total knee arthroplasty (TKA). The aim of this study is to investigate if calprotectin is reliable for PJI diagnosis in cases with accompanying inflammation due to recent surgery, dislocation or implant breakage in primary and revision TKA and THA.

**Methods:**

Thirty-three patients were included in this prospective study between July 2019 and October 2021 (17 patients undergoing surgery < 9 months, 11 dislocations, five implant breakage, respectively). Synovial white blood cell count (WBC), percentage of polymorphonuclear neutrophils (PMC), serum C-reactive protein (CRP) and synovial calprotectin, using a lateral-flow-assay, were analysed. These parameters were tested against a modified European-Bone-and-Joint-Infection-Society (EBJIS) definition with adjusted thresholds to account for the local inflammation. Statistic quality criteria were calculated and compared using a binary classification test.

**Results:**

Seventeen patients were classified as confirmed infections according to the modified EBJIS definition (13 THA and 4 TKA). The calprotectin assay yielded a sensitivity of 0.88 (0.64, 0.99), a specificity of 0.81 (0.54, 0.96), a positive predictive value (PPV) of 0.83 (0.59, 0.96) and a negative predictive value (NPV) of 0.87 (0.60, 0.98).

**Conclusions:**

Even in the presence of local inflammation due to other, non-infectious causes, calprotectin is a reliable diagnostic parameter for the detection of a PJI in primary and revision THA and TKA.

## Introduction

Periprosthetic joint infection (PJI) remains one of the major causes for total hip arthroplasty (THA) and total knee arthroplasty (TKA) revisions [1, 2]. Its consequences are devastating both for the patient and healthcare providers. The costs associated with septic TKA revisions in the USA are estimated to be over US $1.62 billion in 2020 [3]. Therefore, the appropriate and timely diagnosis of PJI is critical for patients’ outcome and reduction of resources required. However, diagnosing low-grade PJI is challenging and relies on pre-and post-operative findings: synovial and serum markers as well as the microbiological, histopathological and imaging analyses have to be considered [2, 4, 5].

**Fig. 1:**
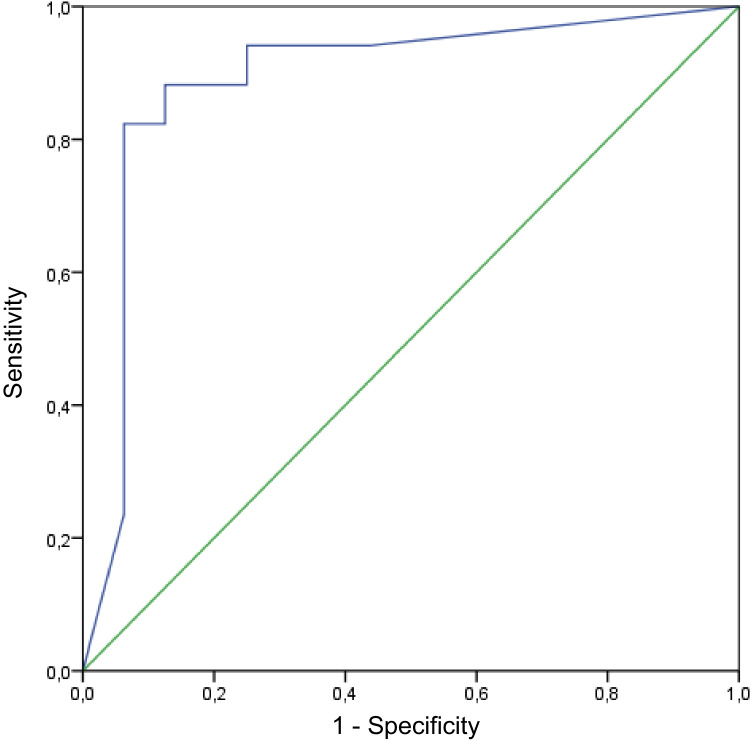
Area under the receiver operating characteristic curve (AUC) of the calprotectin lateral-flow-test, using the modified EBJIS 2021 definition as gold standard. *AUC* = 0.89; EBJIS 2021, European Bone and Joint Infection society definition of periprosthetic joint infection published in 2021

The diagnostic algorithm becomes even more complex if other aseptic causes of local inflammation around the prosthesis are present [4]. Unfortunately, such accompanying inflammations often first raise suspicion of PJI and are the very cause for initiating further diagnostics, i.e. after dislocations or implant breakage (Fig. 1). Similarly, early stages of low-grade PJI may be obscured by the elevation of acute-phase inflammatory markers in the post-operative phase. Recent studies have sought to establish threshold levels for these markers in the early post-operative period, concluding that synovial white blood cell count (WBC) and serum C-reactive protein (CRP) are useful. However, the thresholds suggested change over time as the local inflammation subsides, which may further complicate the diagnostic process [6–9]. Regarding thresholds of these markers after dislocations or implant breakage, the literature is scarce. It appears that common synovial biomarkers like WBC are elevated, but not specific for PJI diagnostics after THA dislocations [10]. In the presence of aseptic local inflammation, the diagnostic value of conventional synovial and serum markers may therefore be impaired and low-grade PJI may be likely overlooked [6, 7].

Thus, the identification of novel biomarkers for these particular cases may be valuable to ascertain or refute the diagnosis of PJI. Recently, synovial calprotectin has been demonstrated as a promising biomarker for PJI in THA and TKA [11, 12]. Calprotectin is a proinflammatory protein that is released from activated granulocytes and macrophages during inflammation [13]. The purpose of this study is to evaluate calprotectin as a biomarker for PJI in cases with accompanying inflammation due to dislocations, implant breakage and recent surgical interventions in TKA and THA revisions.

## Material and methods

**Fig. 2:**
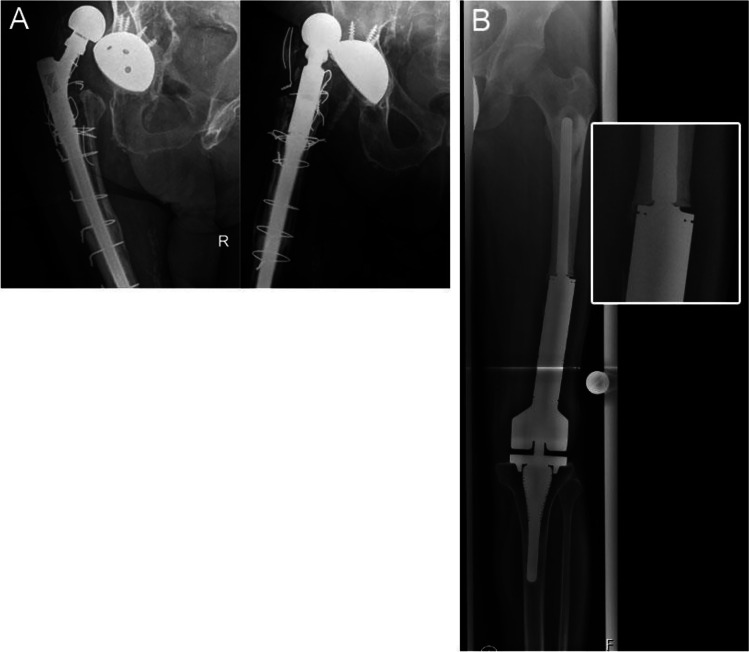
X-rays of dislocated revision THA and implant breakage of distal femur replacement. **A** A 70-year-old patient with dislocation of revision THA. Last dislocation occurred 5 years before index surgery. The implant was revised due to periprosthetic fracture 10 years ago. Primary arthroplasty was performed 11 years ago due to aseptic necrosis of the femoral head. **B** A 30-year-old patient with implant breakage of a distal femur replacement 10 years after treatment for osteosarcoma. The breakage in the junction to the socket is enlarged

We prospectively included all patients undergoing revision arthroplasty for THA and TKA that showed one of the following potential causes of accompanying inflammation between July 2019 and October 2021: 17 patients in the early post-operative phase (13, 3 and 1 patients within 3, 6 and 9 months, respectively) (postoperative group), 11 patients after dislocation (dislocation group) and five patients with implant breakage (implant breakage group). A total of 33 patients were included. Cases missing complete serum and synovial fluid markers and a histopathological evaluation due to incomplete reporting were excluded (*n* = 2). This study was approved by our institutional ethics commission under no. 26/19 S-SR. Informed consent was obtained from all patients. Serum markers and clinical evaluation were performed during the routine pre-operative workup according to our standardized work flow for revision cases. All joints were aspirated intra-operatively under aseptic conditions to measure the WBC and the percentage of polymorphonuclear neutrophils (PMN). Thereafter, the calprotectin lateral-flow-test (Lyfstone AS, Lysaker, Norway) was conducted according to the manufacturer's instructions. After 15 minutes, the test results were photometrically assessed using a smartphone application provided by Lyfstone. Therefore, the test cassette is mounted in a frame that permits detection by the mobile application. The photometric evaluation of the test lines is performed as soon the test is detected with the mobile phone camera (Fig. 2). The concentration of calprotectin is proportional to the colour intensity and can therefore be calculated by the mobile application. The quantitative output of the calprotectin level ranges from 14 to 300 mg/ml. The manufacturer recommends three risk stratification groups to estimate the risk of a PJI: low risk (less than 14 mg/ml), moderate risk (14 to 50 mg/ml) and high risk (more than 50 mg/ml).

To account for the accompanying inflammation in the respective groups, the cut-off values for WBC, PMN and CRP were modified. As the thresholds for diagnosing low-grade PJI are neither validated for the post-operative period nor dislocations or implant breakage, we applied thresholds according to Sukhonthamarn et al. that we thought to be the most suitable. The authors investigated thresholds for PJI and their time-dependent change within 90 days of the index arthroplasty [7]. Regardless of the clinical diagnosis, the cases were defined as septic or aseptic using this modified version of the EBJIS classification of PJI. As a result, a case was classified as a ‘confirmed infection’ if at least one of the following findings was positive: presence of pus around the prosthesis, *WBC* > 6130/µl, *PMN* > 79.5%, microbiological growth in two samples with the same pathogen or in sonication fluid with > 50 CFU/ml, or presence of type II or III membranes according to Morawietz and Krenn et al. [14]. All statistical calculations were performed using SPSS Statistics (Version 22.0. IBM Corporation, Armonk, NY). Values of *α* < 0.05 were considered to indicate statistical significance.

## Results

Nineteen women and 14 men with a mean patient age at revision of 65.1 ± 13.9 years were analysed (21 THA; 12 TKA). According to the modified EBJIS 2021 definition, 17 out of 33 patients were classified as confirmed infections (13 THA and 4 TKA): nine of 17 patients in the early post-operative phase, seven of 11 patients after dislocation and one of five patients with implant breakage.

Regarding the postoperative group, four confirmed infections could be attributed to increased WBC and PMN levels. Three of these four cases also showed positive histology and microbiological growth (*Staphylococcus aureus*, *Staphylococcus warneri* and *Cutibacterium avidum*) and one case showed additionally elevated CRP levels. Four additional confirmed infections were detected by microbiological growth of *Staphylococcus epidermidis* in two instances, *Pseudomonas aeruginosa* and *Cutibacterium acnes*. Of these latter four cases, three had additional positive histology, two showed elevated CRP and one case showed elevated PMN, respectively. One confirmed infection was entirely based on elevated PMN. Regarding the dislocation group, two confirmed infections were defined by elevated WBC, PMN and microbiological growth (both *Staphylococcus epidermidis*). One had increased CRP levels, and the other showed positive histology, in addition. Another four confirmed infections were derived from elevated PMN. Two of these four cases had elevated CRP levels and one case showed increased WBC, respectively. One further case was considered to be infected due to positive histology. One confirmed infection due to positive histology was seen in the implant breakage group. The histological evaluation was not conclusive in 12 cases (6 cases in the post-operative group, 3 cases in the dislocation group and one case in the implant breakage group). The performance of CRP, WBC and PMN levels as well as the performance of the calprotectin lateral-flow-test were statistically measured with the modified post-operative EBJIS 2021 definition serving as the gold standard. The results are demonstrated in Table 1.

**Table 1:** Performance of CRP, WBC, PMN and calprotectin lateral-flow-test. The modified EBJIS 2021 definition served as the gold standard. CI, confidence interval; PPV, positive predictive value; NPV, negative predictive value; EBJIS 2021, European Bone and Joint Infection society definition of periprosthetic joint infection published in 2021

	CRP	WBC	PMN	Calprotectin
Sensivity (95% *CI*)	0.41 (0.18, 0.67)	0.41 (0.18, 0.67)	0.71 (0.44, 0.90)	0.88 (0.64, 0.99)
Specificity (95% *CI*)	0.81 (0.54, 0.96)	1.00 (0.79, 1.00)	1.00 (0.79, 1.00)	0.81 (0.54, 0.96)
PPV (95% *CI*)	0.70 (0.35, 0.93)	1.00 (0.59, 1.00)	1.00 (0.74, 1.00)	0.83 (0.59, 0.96)
NPV (95% *CI*)	0.57 (0.35, 0.77)	0.62 (0.41, 0.80)	0.77 (0.53, 0.92)	0.87 (0.60, 0.98)
Accuracy	0.6	0.7	0.85	0.85

Fifteen out of 17 confirmed infections according to the modified EBJIS 2021 definition were identified using the calprotectin lateral flow assay. The calprotectin lateral flow assay yielded three false positive and two false negative results based on the modified EBJIS 2021 definition. Two false positive cases resulted in failed distal femoral replacements — ten years after osteosarcoma and 12 years after synovial sarcoma. The first case showed failure of the femoral stem. Calprotectin exceeded 300 mg/ml while all other parameters were without pathological findings. The second case showed failure of the constraint mechanism. Calprotectin was measured at 71 mg/dl while all other parameters again remained without pathological finding. The third false positive case refers to recurrent dislocations of a THA with a calprotectin of 61 mg/dl and no further pathological findings.

**Fig. 3:**
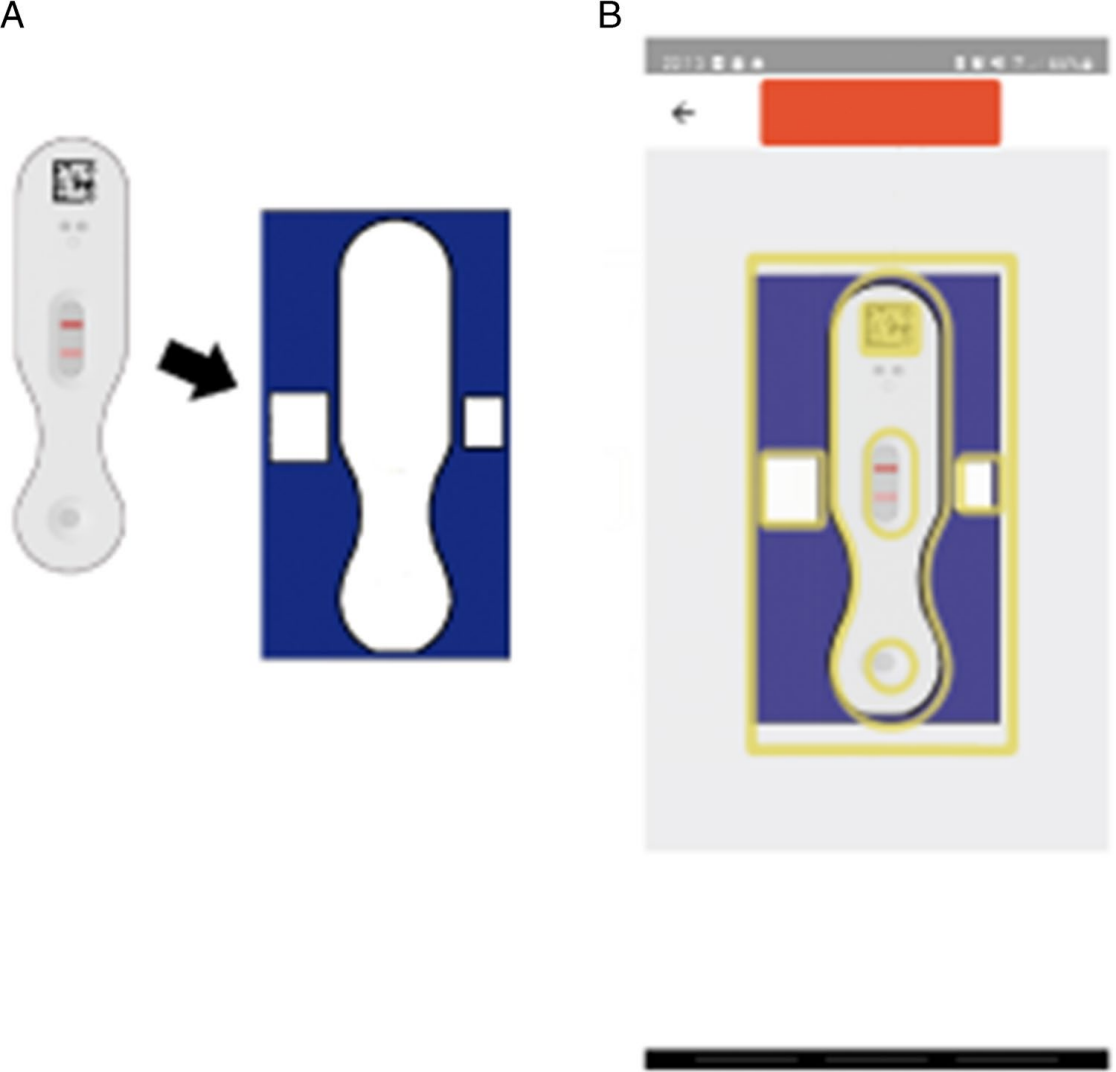
Illustration of the photometric readout by the Lyfstone application. **A** The test cassette is mounted in a frame that permits detection by the mobile application. **B** Illustrated screenshot of the mobile application: the photometric evaluation of the test lines is performed as soon the test is detected with the mobile phone camera by overlapping the frame on the screen (yellow) with the test cassette mounted in the frame (blue). The concentration of calprotectin is proportional to the colour intensity and can therefore be calculated by the application. The illustrations have been modified based on Lyfstone AS, Tromsø, Norway

Both false negative cases were found in the dislocation group. The first false negative case showed a calprotectin level of 42 mg/dl. This case was ruled positive only because of the PMN of 91%. The second false negative case showed a calprotectin level of 14 mg/dl while the WBC was 14,000/µl with a PMN of 83%. The area under the receiver-operation characteristic curve (AUC) for the calprotectin lateral-flow-test was 0.89, with the optimal threshold at 70.5 mg/dl calprotectin with a sensitivity of 0.88 and specificity of 0.88 (Fig. 3). Regarding the subgroup of cases investigated within 3 months postoperatively (*n* = 12), the calprotectin lateral-flow-test yielded excellent results with a sensitivity, specificity, PPV and NPV of 1.00 (0.54, 1.00), respectively.

### Discussion

The most important finding of this study is that calprotectin has an excellent diagnostic value in cases with other possible causes of inflammation that may mimic infection. The diagnostic performance of the calprotectin lateral-flow-test outperformed the results of WBC, PMN and CRP in this study. The performance of the calprotectin test was so accurate that the other markers provided little further benefit. Only the PMN enhanced the diagnostic yield within the scope of this study. Moreover, the diagnostic performance of calprotectin is comparable to the performances of WBC, PMN and CRP with retrospectively determined thresholds in studies focusing on accompanying inflammation in the post-operative period. Kim et al. retrospectively analysed possible PJI in 197 patients with knee arthroplasties three weeks post-operatively and yielded a sensitivity and specificity of 1.0 and 0.99 for WBC with a cut-off set at 11,200/μl and 1.0 and 0.9 for CRP with a cut-off set at 34.9 mg/l, respectively [15]. Similarly, Yi et al. retrospectively investigated 122 THA that underwent revision within the first six weeks post-operatively and reported a sensitivity and specificity of 0.89 and 1.0 for WBC (12,800/µl), 0.81 and 0.9 for PMN (89%) and 0.91 and 0.87 for CRP (93.0 mg/l), respectively [9]. Sukhonthamarn investigated the thresholds for diagnosing PJI within 90 days post-operatively in 197 patients with joint arthroplasties and reported a sensitivity and specificity of 0.91 and 0.83 for WBC (6130/µl), 0.95 and 0.59 for PMN (79.5%) and 0.91 and 0.87 for CRP (38.9 mg/dl), respectively [7]. The thresholds and the diagnostic accuracy of these biomarkers vary and have neither been finally validated for the early post-operative period nor for dislocations or implant breakages. Likewise, the lateral-flow-test for calprotectin has not been validated under these circumstances. Two out of three false positive cases showed elevated calprotectin levels defined as moderate risk for PJI. The inaccuracy of photometrical evaluation of this point of care test must be taken into account in this respect. In contrast, two out of three false positive cases resulted from tumour prosthesis with mechanical failure of the constraint mechanism. It has been demonstrated that metallosis might impair the results of calprotectin lateral-flow-tests [12]. Implant breakage of such megaprostheses may result in debris that triggers joint inflammation in a similar way. Hence, implant breakage may similarly interfere with the calprotectin test.

Interestingly, the diagnostic performance of the calprotectin test was particularly good within three months post-operatively, in which all septic and aseptic cases were correctly identified. Although this finding relates to only 12 cases, it is an incentive to further investigate the performance of calprotectin in PJI diagnostics in this early post-operative period. In general, diagnosing PJI demands a comprehensive understanding of the patient’s medical history, the clinical presentation as well as the understanding of diagnostic algorithms and biomarker evaluation. We therefore conclude that calprotectin may be of value as a further component in the diagnostic algorithm for PJI.

Several authors argued that inflammation markers are a continuum that is subject to temporal changes in septic and aseptic cases [7, 9, 16]. Similarly, we assumed that the thresholds for calprotectin will change over time in the post-operative group and will differ between the other groups as they may trigger different levels of inflammation. The threshold for calprotectin resulting from our ROC-analysis was higher than the thresholds recommended for PJI diagnostics in primary arthroplasties, indicating elevated levels of inflammation in aseptic and septic cases. Applying the lateral-flow-test, however, we could not demonstrate time-dependent changes within the post-operative group nor significant changes between the dislocation, implant breakage and post-operative groups. Although we could demonstrate promising results with this easy-to-use test, more extensive analyses are needed in order to investigate this assumption. We therefore advocate to further investigate the calprotectin levels over time in this context with more accurate quantitative methods like enzyme-linked immunosorbent assays.

This study has several limitations. First, heterogeneous causes of inflammation were included. However, instead of defining thresholds for very specific clinical situations and time periods, we aimed to analyse calprotectin levels in a wide range of cases with inflammation of other causes that may obscure PJI, in order to test for applicability in different settings. Nevertheless, our results are comparable to those who examined calprotectin in homogeneous groups of TKA and THA [11, 12, 17]. Second, the respective groups were limited in size. However, the occurrence of low-grade PJI in the early post-operative stage and in the other groups is rare [9]. More data is required to confirm these results in every group. Therefore, no final conclusions should be drawn regarding the thresholds and especially the implementation of calprotectin in the established diagnostic algorithm, as neither WBC, PMN and CRP nor calprotectin has been validated for this purpose. However, to our knowledge, this is the first study to prospectively investigate the role of calprotectin in respect to aseptic causes of inflammation after total joint arthroplasty. Third, a selection bias may be present in the post-operative and dislocation groups, since not all cases are necessarily revised and subject to a complete diagnostic workup including intraoperative biopsies and sonication. However, as standard procedure in a high-volume, national referral centre for arthroplasty revision surgery, any reasonable suspicion of PJI has been investigated.

In conclusion, the calprotectin lateral flow assay seems to be promising diagnostic test for PJI detection when other causes of inflammation are present. Its diagnostic performance was higher than the performance of WBC, PMN and CRP in this study and is comparable to the performances of these biomarkers in the literature. To define the utility of the calprotectin lateral-flow-test more precisely in this context, however, larger prospective studies are required.
